# Supplementary material for: Cyclic peptides discriminate BCL-2 and its clinical mutants from BCL-XL by engaging a single-residue discrepancy
Source: Nat Commun. 2024 Feb 17;15:1476. doi: 10.1038/s41467-024-45848-1 (PMC10874388; doi:10.1038/s41467-024-45848-1)
Supplement: Supplementary file 1 — Supplementary Information [file 41467_2024_45848_MOESM1_ESM.pdf]

Supplementary Information

Cyclic peptides discriminate BCL-2 and its clinical mutants from BCL-X_L by engaging a single-residue discrepancy

Fengwei Li[1,7,*], Junjie Liu[2,7], Chao Liu[1,7], Ziyan Liu[2], Xiangda Peng[3], Yinyue Huang[1], Xiaoyu Chen[1], Xiangnan Sun[1], Sen Wang[1], Wei Chen[4], Dan Xiong[5], Xiaotong Diao[1], Sheng Wang[3], Jingjing Zhuang[1,6], Chuanliu Wu[2,*], Dalei Wu[1,*]

[1]Helmholtz International Lab, State Key Laboratory of Microbial Technology, Shandong University, Qingdao 266237, China.

[2]The MOE Key Laboratory of Spectrochemical Analysis and Instrumentation, State Key Laboratory of Physical Chemistry of Solid Surfaces, Department of Chemistry, College of Chemistry and Chemical Engineering, Xiamen University, Xiamen 361005, China.

[3]Shanghai Zelixir Biotech Company Ltd., Shanghai 200030, China.

[4]Shanghai Immune Therapy Institute, Shanghai Jiao Tong University School of Medicine-Affiliated Renji Hospital, Shanghai 200127, China.

[5]Xiamen Lifeint Technology Company Ltd., Xiamen 361005, China.

[6]Marine College, Shandong University, Weihai 264209, China

[7]These authors contributed equally: Fengwei Li, Junjie Liu, Chao Liu

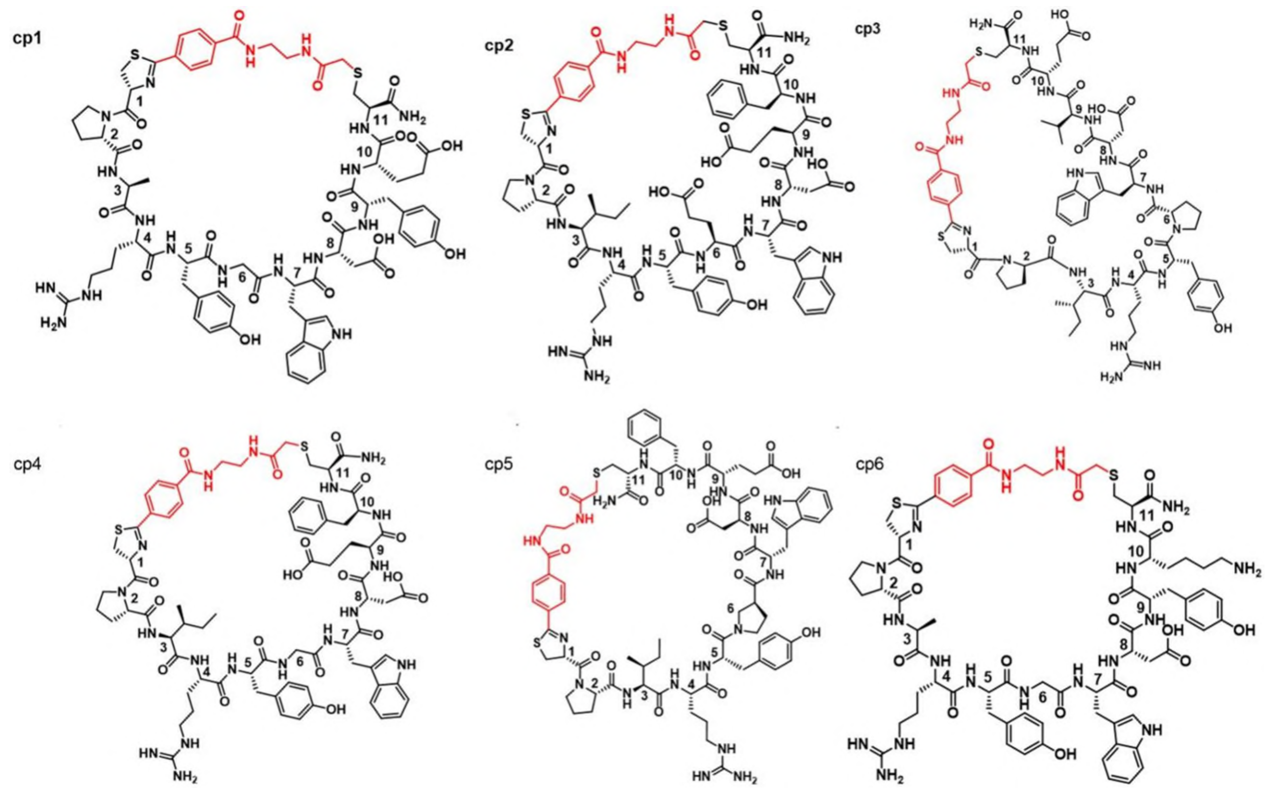

Supplementary Figure 1 Chemical structures of CPs used in this study. Linker c-ADT is colored in red.

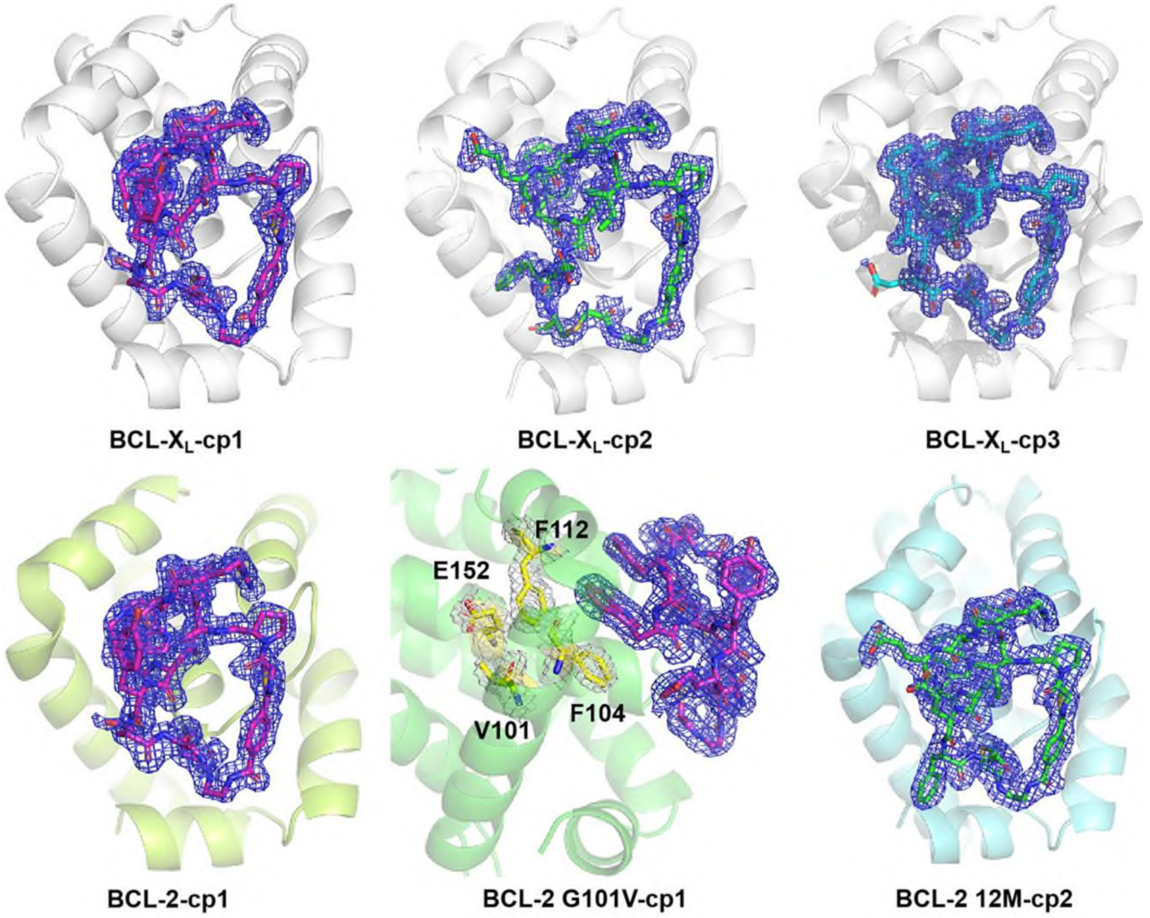

Supplementary Figure 2 The 2Fo−Fc density maps of the CPs each bound to BCL-X_L, BCL-2 or BCL-2 G101V mutant. All density maps are contoured at 1.0 σ, with maps of CPs and residues undergoing conformational changes (V101, F104, F112 and E152) in BCL-2 G101V colored in blue and white, respectively.

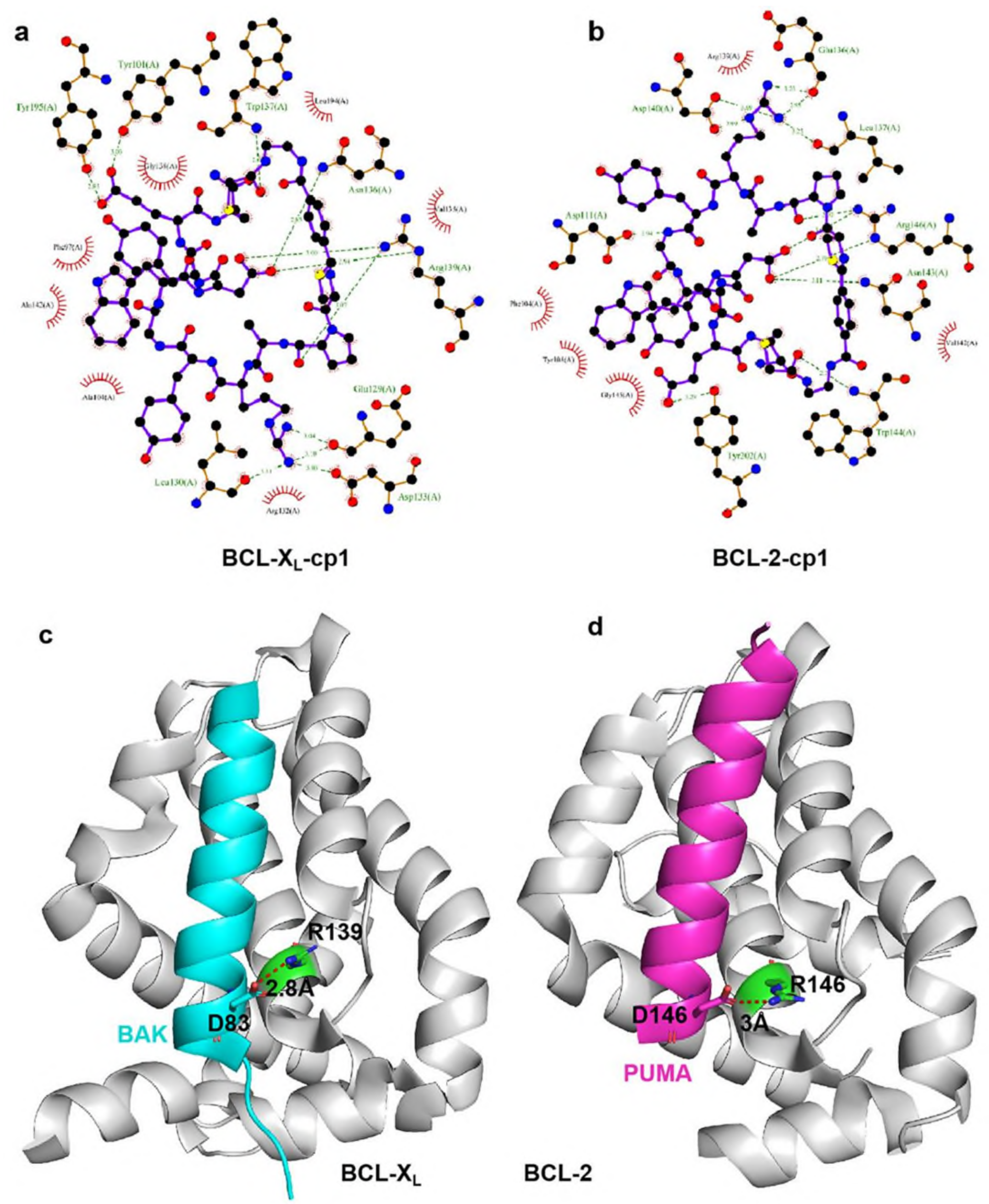

Supplementary Figure 3 Key residues of BCL-X$_L$ and BCL-2 around cp1 and a special interacting mode of BH3-only peptides binding to BCL-X$_L$ or BCL-2. a, b. Key protein residues around cp1 in the BCL-2-cp1 (**a**) and BCL-X$_L$-cp1 (**b**) complexes. **c, d.** The conformation of BAK (**c**) and PUMA (**d**) in complex with BCL-X$_L$ (**c**, PDB ID: 5FMK) and BCL-2 (**d**, PDB ID: 6QG8), respectively. The D83 of BAK and R139 of BCL-X$_L$ shown as sticks in cyan and green, while the D146 of PUMA and R146 of BCL-X$_L$ are shown as sticks in magenta and green, respectively.

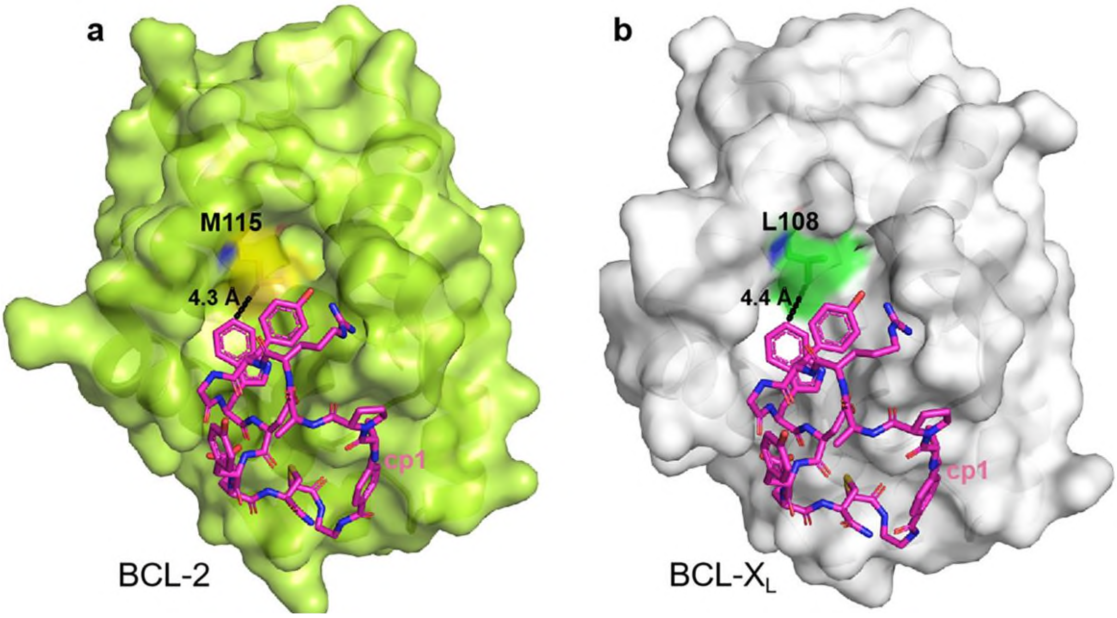

Supplementary Figure 4 The comparison of another pair of discrepant residues between BCL-X$_L$ and BCL-2. **a, b.** The M115 residue of BCL-2 (**a**) and the L108 residue of BCL-X$_L$ (**b**) are both highlighted in the protein-cp1 complexes. The minimum distances between these two residues and cp1 (magenta) are shown as black dotted lines.

a

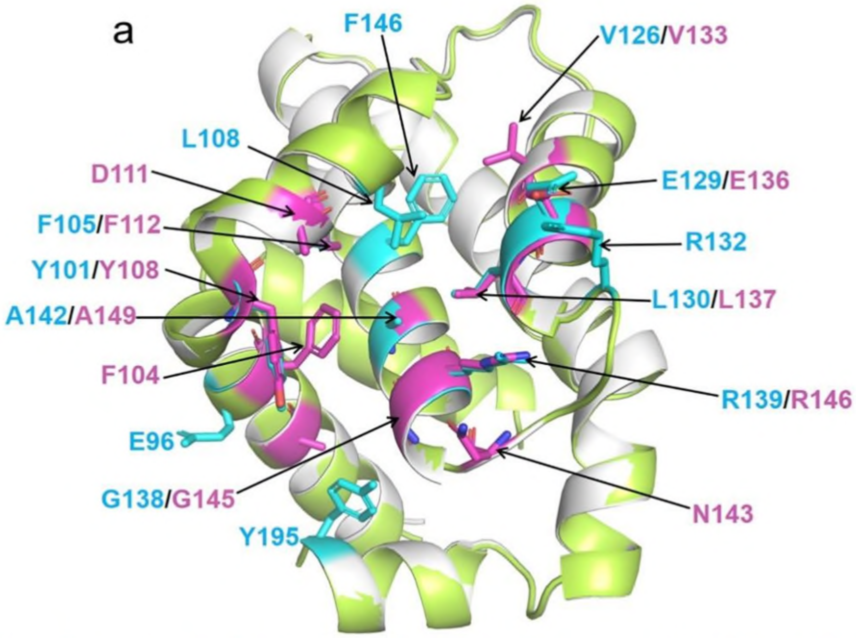

b

BCL-X_L	A93	F97	Y101	A104	F105	L108	V126	E129	L130	R132	N136	G138	R139	A142	F146	Y195
BCL-2	A100	F104	Y108	D111	F112	M115	V133	E136	L137	R139	N143	G145	R146	A149	F153	Y202

Supplementary Figure 5 Comparative analysis of key amino-acid residues of BCL-2 and BCL-X_L interacting with different inhibitors. All available structures of BCL-2 (PDB ID: 2xa0, 4aq3,4lvt, 4lxd, 4man, 4ieh, 5jsn, 5fcg, 5vau, 5vay, 5agw, 5agx, 6o0k, 6o0o, 6qg8, 6qgg) and BCL-X_L (PDB ID: 1bxl, 1g5j, 1pql, 2yxj, 2p1l, 2bzw,2yjl, 3pl7, 3qkd, 4ehr, 4alu, 3spf, 2yq6, 2yq7, 4bpk, 3zlr, 4qvx, 4tuh, 4c5d, 4qve, 4qvf, 5c3g, 5fmj, 5fmk, 4z9v, 6igq, 6dco, 6dcn, 6hjl, 7jgv, 7lh7, 6st2, 6uvc, 6uvd, 6uve) in the PDB database are included. **a.** Key residues of BCL-2 (colored in magenta) and BCL-X_L (colored in cyan) contacting to all inhibitors within 5 Å distance are labeled. The overall structures of BCL-2 and BCL-X_L are shown as cartoon in limon and white. **b.** Protein residues within 5 Å around cp1 in the BCL-X_L-cp1 and BCL-2-cp1 complexes. Cyan and magenta fonts represent conserved key residues around all inhibitors within 5 Å in BCL-X_L and BCL-2, respectively.

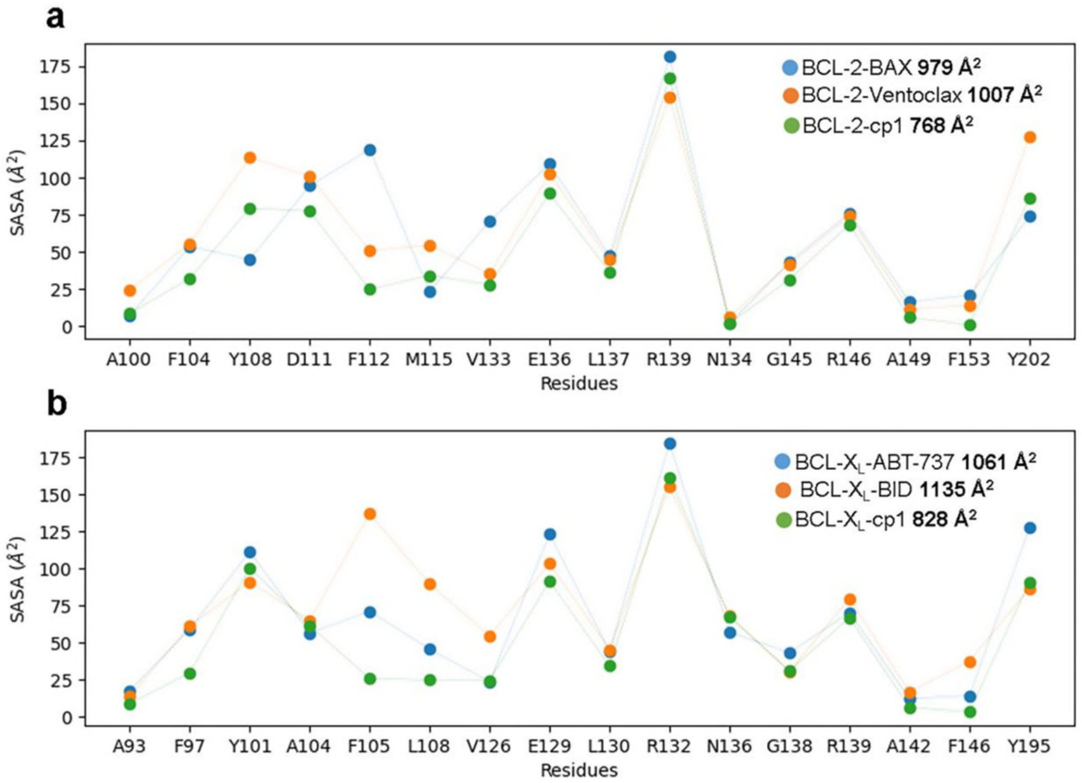

Supplementary Figure 6 The solvent-accessible surface area (SASA) of key amino acids on BH3-only binding surface of BCL-2 (a) and BCL-X_L (b) in different complexes.

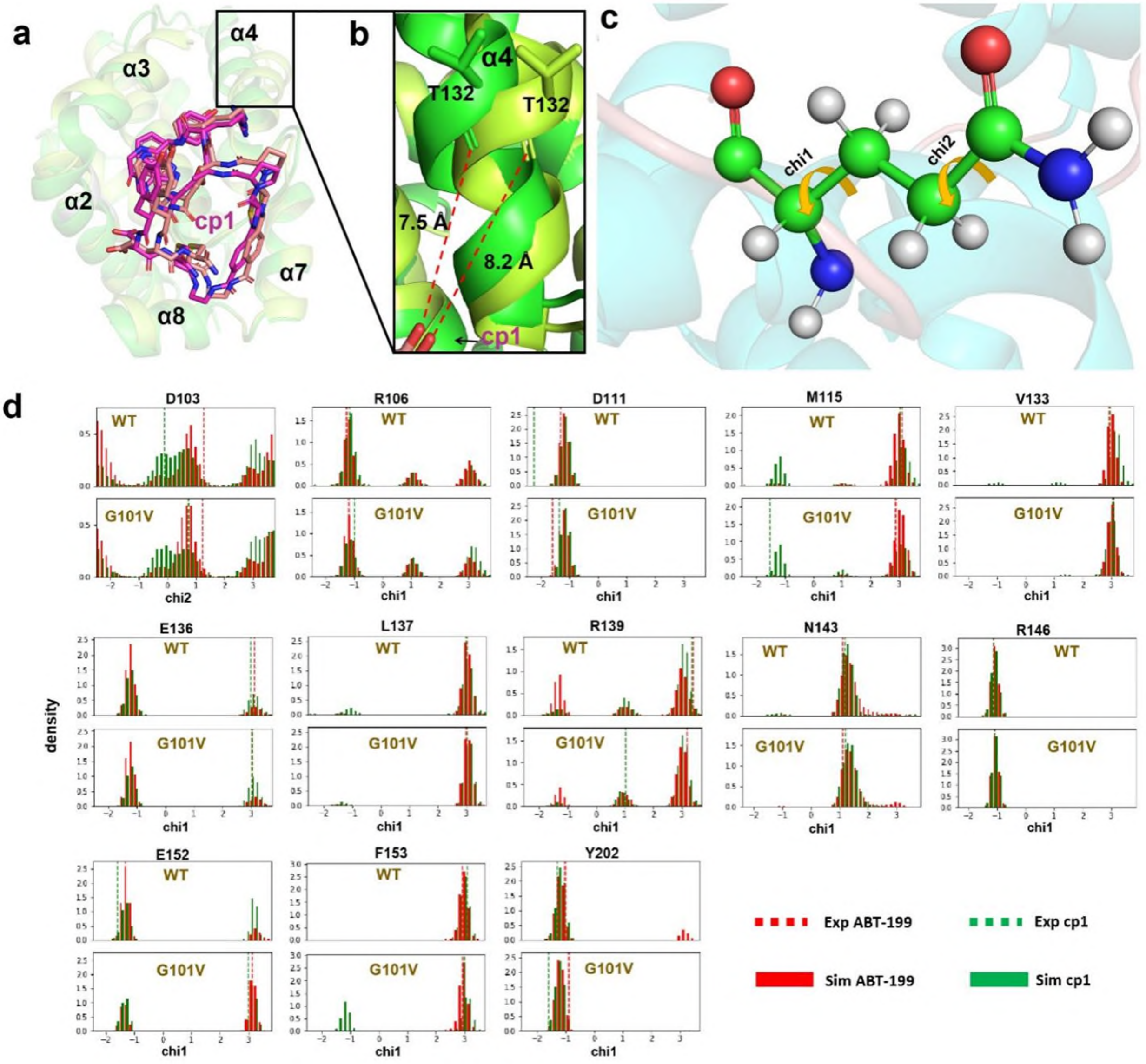

Supplementary Figure 7 The structural mechanism of cp1 overcoming BCL-2 G101V mutation. a. Structural comparison of cp1-bound BCL-2 WT and G101V mutant. The overall structures of WT and G101V are shown as cartoon in limon and green; cp1 molecules binding to WT and G101V are shown as sticks in magentas and brown, respectively. **b.** The deviation region (N-terminus) of α4 helix each in cp1-bound BCL-2 WT and G101V mutant, shown as cartoon in white and limon. **c.** Schematic for chi1 and chi2 angles of amino-acid residues. **d.** Comparison of the chi angle distribution of residues from BCL-2 WT or G101V mutant in venetoclax-bound state (red) or cp1-bound state (green). Dotted lines represent the chi angle in experimental (Exp) status (in crystal structures), while solid lines represent the distribution of chi angles in stimulation (Sim).

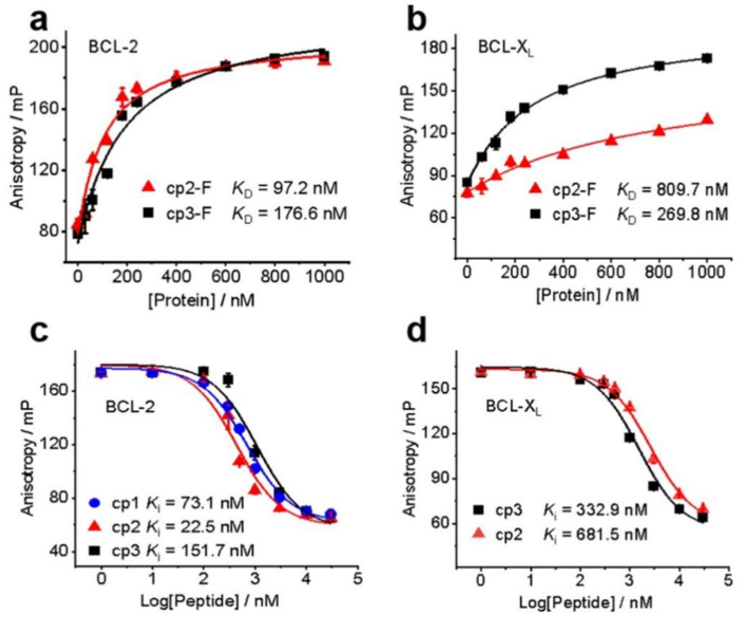

Supplementary Figure 8 Binding affinities of CPs upon BCL-2 or BCL-X_L proteins as measured by the fluorescence polarization assay. a, b. Binding of cp2-F/cp3-F to BCL-2 (**a**) or BCL-X_L (**b**). **c.** Binding of cp1/cp2/cp3 to BCL-2 by competition with cp2-F. **d.** Binding of cp2/cp3 to BCL-X_L by competition with cp3-F. Each data point represents the mean ± s.d. for 3 replicated measurements.

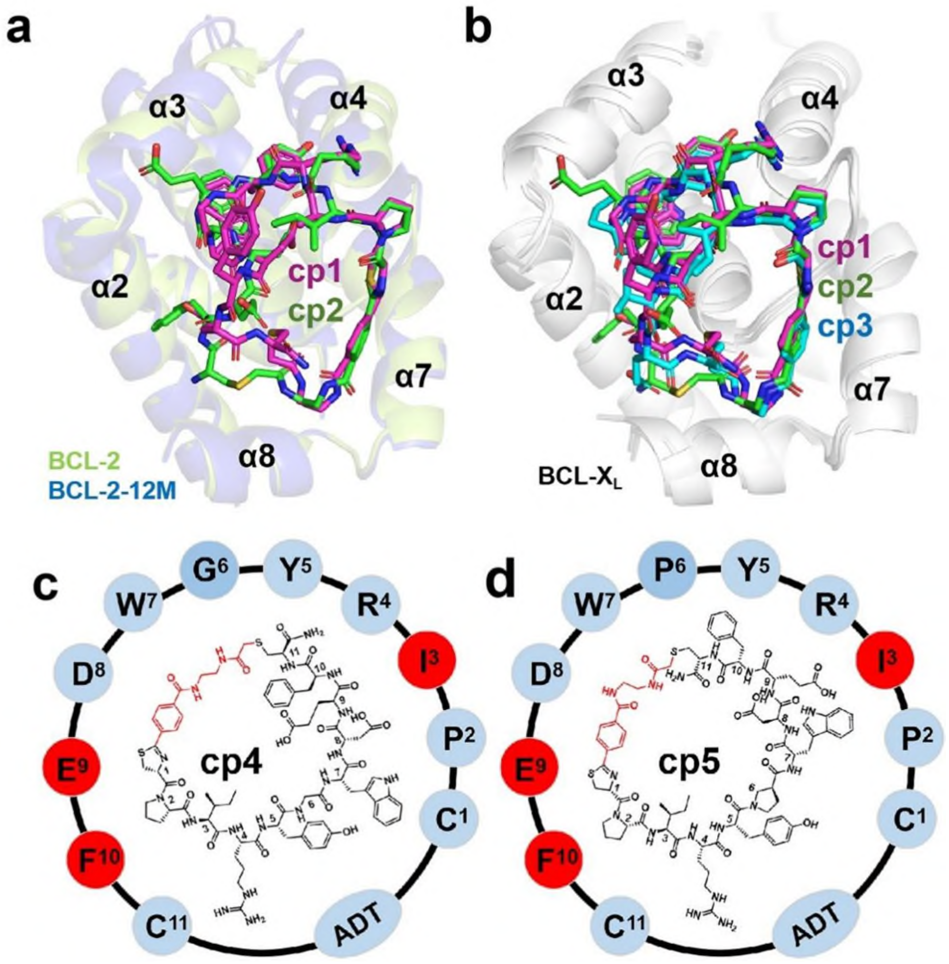

Supplementary Figure 9 Structure-based design for new CPs. a. The overall structure comparison between BCL-2-cp1 and BCL-2-12M-cp2. BCL-2 and BCL-2-12M are shown as carton in light green and light blue, while cp1 and cp2 are colored in magenta and green. **b.** Comparison of the overall structures of BCL-X_L proteins each in complex with cp1, cp2 and cp3. The three CPs, cp1, cp2 and cp3 are shown as sticks in magenta, green and cyan, respectively. **c,d.** Structures of newly designed CPs, cp4 (**c**, derived from cp2 by replacing 6E with 6G) and cp5 (**d**, derived from cp2 by replacing 6E with 6P).

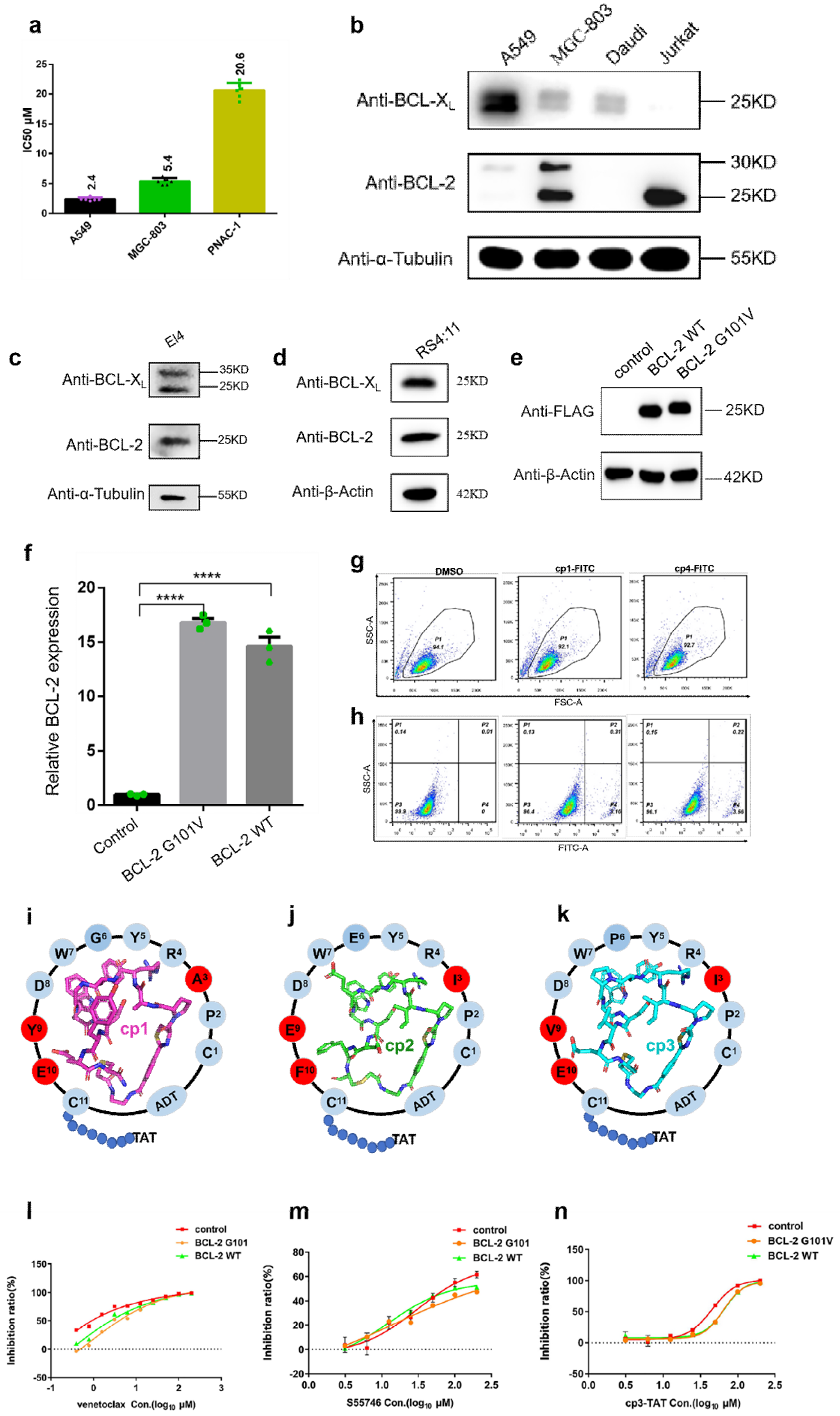
a
IC50 μM
2.4
5.4
20.6
A549
MGC-803
PNAC-1
b
A549
MGC-803
Daudi
Jurkat
Anti-BCL-XL
Anti-BCL-2
Anti-α-Tubulin
25KD
30KD
25KD
55KD
c
El4
Anti-BCL-XL
Anti-BCL-2
Anti-α-Tubulin
35KD
25KD
25KD
55KD
d
RS4:11
Anti-BCL-XL
Anti-BCL-2
Anti-β-Actin
25KD
25KD
42KD
e
control
BCL-2 WT
BCL-2 G101V
Anti-FLAG
Anti-β-Actin
25KD
42KD
f
Relative BCL-2 expression
****
****
Control
BCL-2 G101V
BCL-2 WT
g
DMSO
cp1-FITC
cp4-FITC
SSC-A
FSC-A
h
SSC-A
FITC-A
i
cp1
TAT
ADT
j
cp2
TAT
ADT
k
cp3
TAT
ADT
l
control
BCL-2 G101
BCL-2 WT
Inhibition ratio(%)
venetoclax Con.(log10 μM)
m
control
BCL-2 G101
BCL-2 WT
Inhibition ratio(%)
S55746 Con.(log10 μM)
n
control
BCL-2 G101V
BCL-2 WT
Inhibition ratio(%)
cp3-TAT Con.(log10 μM)

Supplementary Figure 10 Functional mechanism of CPs. a. IC50 values of venetoclax against A549, MGC-803 and PANC-1 cells, measured after 48 h treatment by a CellTiter-Glo assay and calculated using Prism 7.0 by a nonlinear-fit method. Data are presented as mean ± s.d.; n= 6 biological replicates. **b.** Western blot detecting the protein expression levels of BCL-2 and BCL-X_L in A549, MGC-803, Daudi and Jurkat cells. **c-d.** Western blot detecting the protein expression levels of BCL-2 and BCL-X_L in El4 cells (**c**) and RS4:11 cells (**d**). **e.** Western blot verifying the protein expression levels of exogenous BCL-2 in normal A549 cells (control) and those stably overexpressing BCL-2 WT or G101V mutant. **f.** qPCR results testing the overexpression of BCL-2 WT or G101V mutant in A549 stable cells. Data are presented as mean ± s.d.; n= 6 biological replicates; one-way ANOVA with Dunnett's multiple comparisons test. **g.** Cell viability assay of Jurkat cells after 48 h treatment with DMSO, cp1-FITC, cp6-FITC, respectively. P1 represents the percentage of active cells. **h.** FITC signal tests after 48 h treatment with DMSO, cp1-FITC, cp6-FITC, respectively. P4 represents cells with the FITC signal. **i-k.** Structures of cp1-TAT, cp2-TAT and cp3-TAT. The red shade indicates the residues not directly involved in the interactions to proteins. **l-n,** Viability of original A549 cells (control), BCL-2 WT overexpressing cells, and BCL-2 G101V overexpressing cells treated with various concentrations of venetoclax (**l**), S55746 (**m**) and cp3-TAT (**n**) measured after 48 h by a CellTiter-Glo assay. Each data point represents the mean ± s.d. for 6 biological replicates.

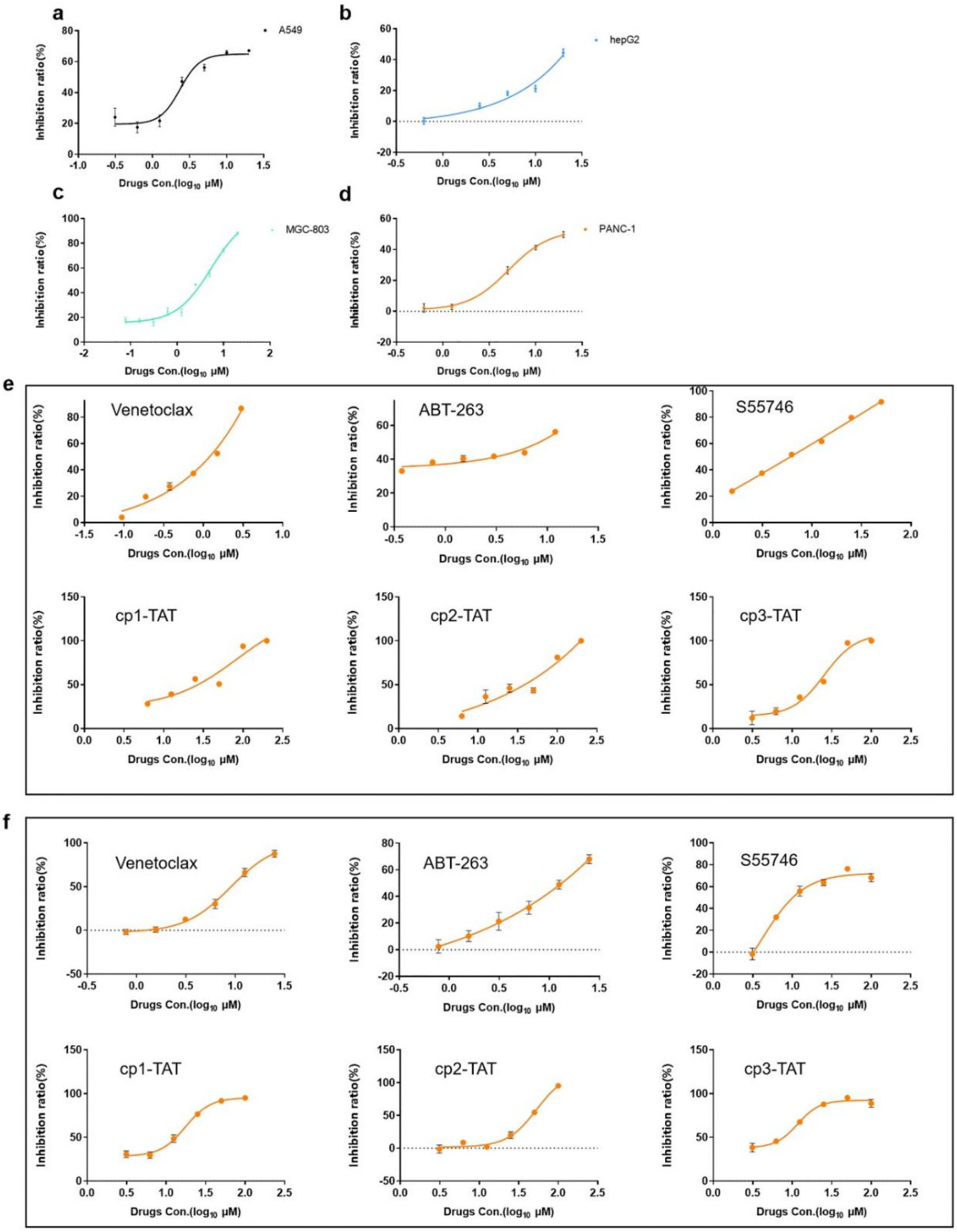

Supplementary Figure 11 IC50 values of different inhibitors against different cell lines. a-d. Viability of A549 (**a**), HepG2 (**b**), MGC-803 (**c**) and PANC-1 (**d**) cells treated with various concentrations of ABT-199 (venetoclax), and measured after 48 h by a CellTiter-Glo assay. **e, f.** Viability of Jurkat cells (**e**) and Daudi cells (**f**) treated with various concentrations of venetoclax, ABT-263, S55746, cp1-TAT, cp2-TAT and cp3-TAT respectively, and measured after 48 h by a CellTiter-Glo assay. Each data point represents the mean ± s.d. for 6 biological replicates.

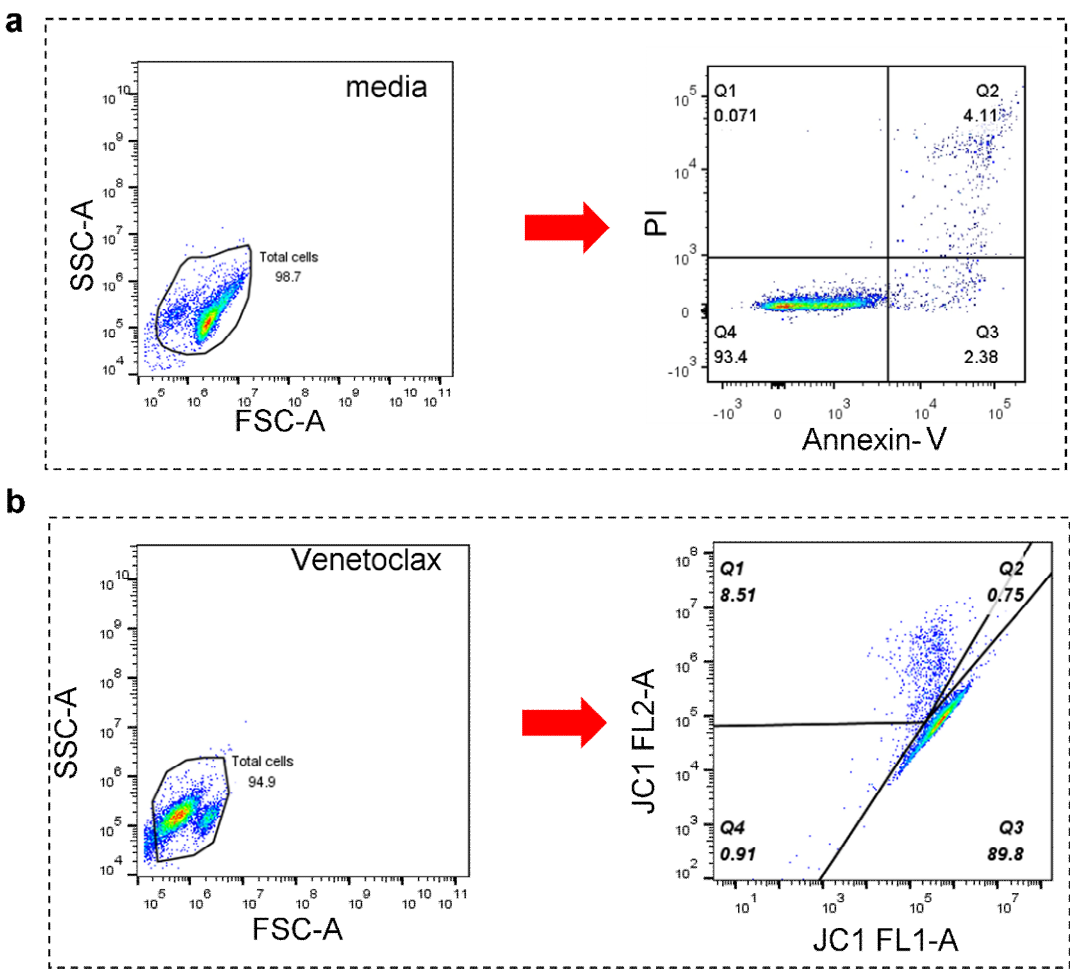

Supplementary Figure 12 FACS sequential gating strategies. a. Based on Jurkat cells' distribution characteristics of FSC-SSC (dead cells are distributed on the left and live cells on the right), total cells were first gated to remove cell fragments and adherent cells. Then Q1-Q4 regions were gated based on the Annexin V-FITC/PI staining distribution, with the results shown in Fig. 6a. **b.** Based on Jurkat cells' distribution characteristics of FSC-SSC (dead cells are distributed on the left and live cells on the right), total cells were first gated to remove cell fragments and adherent cells. Then Q1/Q3 regions were gated based on JC1 staining distribution. Cells with decreased $\Delta\Psi$m emit green fluorescence after treatment with JC1 and distribute in the Q3 region, while normal cells treated by JC1 emit red fluorescence and distribute in the Q1 region. Corresponding results are shown in Fig. 6c.

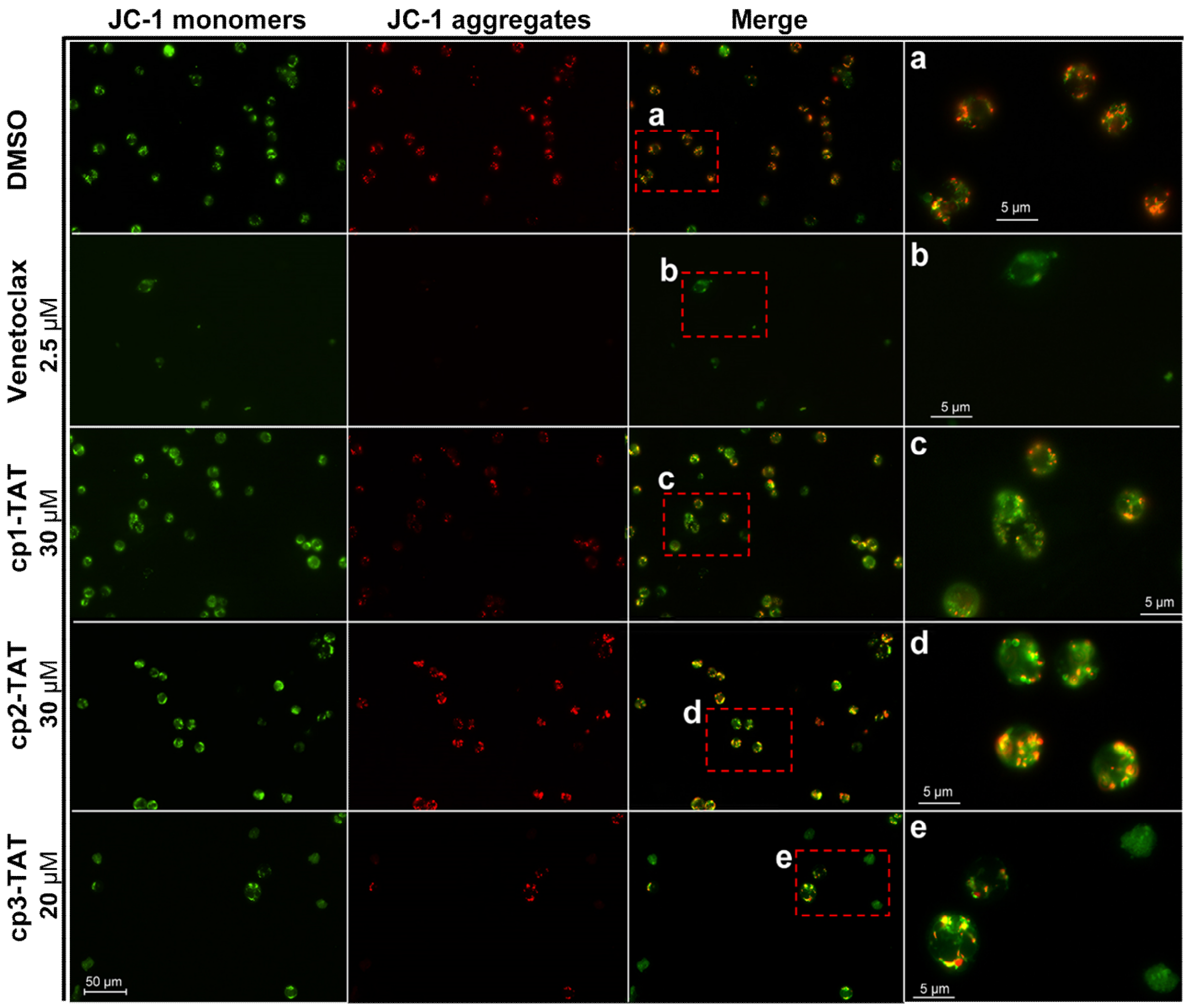

Supplementary Figure 13 The mitochondrial membrane potential (mtΔΨ) analysis of Jurkat cells. Immunofluorescence staining of Jurkat cells after 24 h treatment with different inhibitors as indicated on the left in the figure. Green, JC-1 monomer; red, JC-1 aggregate; scale bar, 50 µm for the left three columns and 5 µm for the enlarged right column (a-e), respectively.

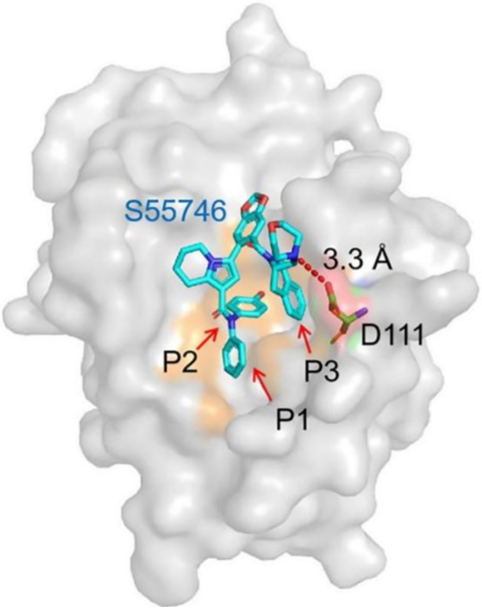

Supplementary Figure 14 The complex structure of S55746-bound BCL-2. The overall structure of BCL-2 is shown as surface in gray (PDB ID:6O0O), and S55746 is shown as sticks in cyan. S55746 binds into the P1, P2, P3 pockets of BCL-2.

Supplementary Table 1 Information about peptides used in the study

Compound	Peptide sequence	Chemical formula	MALDI(M+H)$^+$	
			Cald.	Found
cp1	CPARYGWDYEC	$C_{72}H_{88}N_{18}O_{19}S_2$	1573.599	1573.500
cp1-F	CPARYGWDYECGK(FITC)	$C_{101}H_{114}N_{22}O_{26}S_3$	2147.752	2148.612
cp1-TAT	CPARYGWDYECGSGSRKKRRQRRR	$C_{135}H_{208}N_{52}O_{35}S_2$	3182.561	3182.726
cp2	CPIRYEWDEFC	$C_{78}H_{98}N_{18}O_{20}S_2$	1671.672	1671.791
cp2-F	CPIRYEWDEFCGK(FITC)	$C_{107}H_{124}N_{22}O_{27}S_3$	2245.825	2245.692
cp2-TAT	CPIRYEWDEFCGSGSRKKRRQRRR	$C_{141}H_{218}N_{52}O_{36}S_2$	3280.635	3280.928
cp3	CPIRYPWDVEC	$C_{74}H_{98}N_{18}O_{18}S_2$	1591.683	1591.64
cp3-F	CPIRYPWDVECGK(FITC)	$C_{103}H_{124}N_{22}O_{23}S_3$	2165.835	2165.854
cp3-TAT	CPIRYPWDVECGSGSRKKRRQRRR	$C_{137}H_{218}N_{52}O_{34}S_2$	3200.645	3200.824
cp6	CPARYFWDYKC	$C_{73}H_{93}N_{19}O_{17}S_2$	1572.652	1572.609
cp6-F	CPARYFWDYKCGK(FITC)	$C_{102}H_{118}N_{22}O_{25}S_3$	2147.788	2147.852
cp4	CPIRYGWDEFC	$C_{75}H_{94}N_{18}O_{18}S_2$	1599.651	1599.872
cp5	CPIRYPWDEFC	$C_{78}H_{98}N_{18}O_{18}S_2$	1639.683	1639.673
TAT	GSGSRKKRRQRRR	$C_{63}H_{123}N_{35}O_{16}$	1626.997	1626.906
cp4-TAT	CPIRYGWDEFCGSGSRKKRRQRRR	$C_{138}H_{214}N_{52}O_{34}S_2$	3208.6134	3209.03
cp5-TAT	CPIRYPWDEFCGSGSRKKRRQRRR	$C_{141}H_{218}N_{52}O_{34}S_2$	3248.6447	3249.567
cp1-TAT-8E	CPARYGWDYECGSGSRKKRRQRRR EEEEEEEE	$C_{175}H_{264}N_{60}O_{59}S_2$	4214.9022	4217.123
cp2-TAT-8E	CPIRYEWDEFCGSGSRKKRRQRRR EEEEEEEE	$C_{181}H_{274}N_{60}O_{60}S_2$	4312.9753	4315.057
cp3-TAT-8E	CPIRYPWDVECGSGSRKKRRQRRR EEEEEEEE	$C_{177}H_{274}N_{60}O_{58}S_2$	4232.9855	4234.816

Supplementary Table 2 Crystallographic data collection and refinement statistics

	cp1-bound BCL-X_L	cp2- bound BCL-X_L	cp3-bound BCL-X_L	cp1-bound BCL-2	cp1-bound BCL2-G101V	cp2-bound BCL-2-12M
**Data collection**						
Space group	$C222_1$	$C222_1$	$C222_1$	$C222_1$	$P2_1$	$C222_1$
Cell dimensions						
a, *b*, *c* (Å)	69.80, 99.37, 51.36	68.43, 100.48, 51.55	69.52 99.87 51.20	65.60, 99.61, 52.46	37.73 50.05 48.69	104.76 104.79 111.53
α, β, γ (°)	90.0, 90.0, 90.0	90.0, 90.0, 90.0	90.0, 90.0, 90.0	90.0, 90.0, 90.0	90.0, 107.58, 90.0	90.0, 90.0, 90.0
Resolution (Å)	50.0-2.0 (2.03-2.0)*	50.0-1.9 (1.93-1.9)	50.0-1.40 (1.42-1.40)	50.0-2.1 (2.14-2.1)	50.0-1.85 (1.88-1.85)	50-2.25 (2.29-2.25)
*R*sym or *R*merge	0.073 (0.663)	0.152 (0.492)	0.136 (0.596)	0.105 (0.448)	0.087 (0.342)	0.104 (0.432)
I / σI	34.6 (3.3)	16.2 (5.7)	17.1 (3.0)	24.3 (5.5)	11.7 (1.86)	31.8 (13)
Completeness (%)	98.2 (95.7)	99.9 (99.9)	99.4 (97.4	100 (100)	92.1 (71.9)	100 (100)
Redundancy	12.8 (11.7)	12.6 (12.8)	12.5 (10.4)	12.5 (10.5)	2.9 (2.5)	22.8 (22.3)
**Refinement**						
Resolution (Å)	2.0	1.9	1.4	2.1	1.85	2.25
No. reflections	11034	13311	32773	9132	10965	29689
R_{work} / R_{free}	0.186/0.242	0.175/0.193	0.158/0.181	0.220/0.276	0.191/0.243	0.177/0.194
No. atoms						
Protein	1135	1130	1133	1110	1159	2242
Ligand/ion	111	117	111	111	111	234
Water	74	95	177	87	109	234
B-factors	31.3	22.49	17.53	27.55	30.72	35.18
Protein	30.5	21.76	15.74	26.95	28.56	35.2
Ligand/ion	34.5	23.68	15.57	27.86	21.46	27.76
Water	39.1	29.66	30.06	34.81	38.24	42.16
R.m.s.deviations						
Bond lengths (Å)	0.011	0.012	0.018	0.010	0.010	0.015
Bond angles (°)	1.932	1.875	2.217	1.869	1.884	2.032

One crystal was used for each structure.

*Values in parentheses are for highest-resolution shell.

Supplementary Table 3 Abundant peptide sequences from the new phage-based library screening against BCL-X_L

Sequences											Abundance
C	P	I	R	Y	P	W	D	V	E	C	7
C	P	E	R	Y	P	W	D	S	Y	C	4
C	P	V	R	Y	P	W	D	E	E	C	3
C	P	I	R	Y	P	W	D	E	R	C	2
C	P	I	R	Y	P	W	D	T	E	C	2
C	P	E	R	Y	P	W	D	I	Y	C	1
C	P	E	R	Y	P	W	D	V	P	C	1
C	P	I	R	Y	P	W	D	D	E	C	1
C	P	I	R	Y	P	W	D	E	A	C	1
C	P	I	R	Y	P	W	D	E	R	C	1
C	P	I	R	Y	P	W	D	E	S	C	1
C	P	I	R	Y	P	W	D	S	E	C	1
C	P	V	R	Y	P	W	D	E	P	C	1
C	P	V	R	Y	P	W	D	E	Q	C	1
C	P	V	R	Y	P	W	D	L	E	C	1
C	P	V	R	Y	P	W	D	V	E	C	1
C	P	Y	R	Y	Q	W	D	H	F	C	1

Blue letters represent unchanged key residues (same as cp1) in screening a new phage-based library; black letters represent randomly selected residues; and red letters highlight the very conserved proline at the 6th position of CPs.

Supplementary Table 4 Key residues within 5 Å around CPs in the protein-CP complex structures

BCL-X_L-cp1	BCL-X_L-cp2	BCL-X_L-cp3	BCL-2-cp1	BCL2/G101V-cp1	BCL2/12M-cp2
	A93				
	E96				
F97	F97	F97	F104	F104	F104
	R100		R107	R107	R107
Y101	Y101	Y101	Y108	Y108	Y108
	R103				R110
A104	A104	A104	D111	D111	D111
	F105	F105	F102	F102	F102
L108	L108	L108	M115	M115	M115
E129	E129	E129	E136	E136	E136
L130	L130	L130	L137	L137	L137
F131	F131	F131	F138	F138	F138
R132	R132	R132	R139	R139	R139
D133	D133	D133	D140	D140	D140
G134	G134	G134	G141	G141	G141
V135	V135	V135	V142	V142	V142
N136	N136	N136	N143	N143	N143
W137	W137	W137	W144	W144	W144
G138	G138	G138	G145	G145	G145
R139	R139	R139	R146	R146	R146
	V141				
A142	A142	A142	A149	A149	A149
W181	W181	W181	W188	W188	W188
	N185	N185	N192	N192	N192
L194	L194	L194	L201		L201
Y195	Y195	Y195	Y202	Y202	Y202

Supplementary Table 5 **Binding affinities of CPs-TAT to BCL-2 and BCL-X_L.**

CPs	BCL-2 K_D (μM)	BCL-X_L K_D (μM)
cp1-TAT-8E	0.17 ± 0.03	1.17± 0.07
cp2-TAT-8E	0.14 ± 0.05	0.13 ± 0.009
cp3-TAT-8E	0.07 ± 0.01	0.02 ± 0.001

CPs-TAT-8E, to facilitate SPR detection, 8 negatively charged amino acids glutamic (E) were added at the C-terminus of TAT.

Supplementary Table 6 Comparation of key residues near the BH3-only binding interface of anti-apoptotic proteins within the BCL-2 family.

BCL-2	BCL-X_L	BCL-w	MCL-1	BFL-1
F104	F97	F53	V220	V44
R106	R100	R56	N223	E47
Y108	Y101	F57	H224	K46
D111	A104	T60	A227	N51
F112	F105	F61	F228	V48
M115	L108	L64	M231	L52
E136	E129	E85	H252	K77
L137	L130	L86	V253	E78
F138	F131	F87	F254	F79
R139	R132	Q88	D256	E80
D140	D133	G89	G257	D81
G141	G134	G90	V258	I83
V142	V135	P91	T268	I84
N143	N136	N92	N260	N85
W144	W137	W93	W263	W86
G145	G138	G94	G264	G87
R146	R139	R95	R265	R88
A149	A142	A98	T266	T91
W188	W181	W137	W305	W133
N192	N185	S141	Q309	N137
L201	L194	L150	F318	K147
Y202	Y195	Y151	F319	F148

Light blue color represents conserved residues as compared to BCL-2, while yellow color represents those not conserved.

Supplementary Table 7 Simulation parameters of torsion based HREX

Replica id	Scale factor	Effective temperature (K)
0	1.000	300.0
1	0.950	315.8
2	0.901	333.0
3	0.856	350.5
4	0.812	369.5
5	0.773	388.1
6	0.735	408.2
7	0.700	428.6
