# Supplementary material for: Cyclic peptides discriminate BCL-2 and its clinical mutants from BCL-XL by engaging a single-residue discrepancy
Source: Nat Commun. 2024 Feb 17;15:1476. doi: 10.1038/s41467-024-45848-1 (PMC10874388; doi:10.1038/s41467-024-45848-1)
Supplement: Supplementary file 3 — Description of Additional Supplementary Files [file 41467_2024_45848_MOESM3_ESM.pdf]

Description of Additional Supplementary Files

File Name: Supplementary Data 1
Description: MD simulation parameters. The force fields for cp1, venetoclax and S55746 are stored in the cp1-ff.zip, venetoclax-ff.zip and S55746-ff.zip, respectively. The script used to perform the simulations is provided in REMD.py.
