# Supplementary material for: Cyclic peptides discriminate BCL-2 and its clinical mutants from BCL-XL by engaging a single-residue discrepancy
Source: Nat Commun. 2024 Feb 17;15:1476. doi: 10.1038/s41467-024-45848-1 (PMC10874388; doi:10.1038/s41467-024-45848-1)
Supplement: Supplementary file 5 — Reporting Summary [file 41467_2024_45848_MOESM5_ESM.pdf]

nature portfolio

Corresponding author(s): Dalei Wu

Last updated by author(s): Jan 22, 2024

Reporting Summary

Nature Portfolio wishes to improve the reproducibility of the work that we publish. This form provides structure for consistency and transparency in reporting. For further information on Nature Portfolio policies, see our Editorial Policies and the Editorial Policy Checklist.

Statistics

For all statistical analyses, confirm that the following items are present in the figure legend, table legend, main text, or Methods section.

n/a	Confirmed	
☐	☒	The exact sample size (*n*) for each experimental group/condition, given as a discrete number and unit of measurement
☐	☒	A statement on whether measurements were taken from distinct samples or whether the same sample was measured repeatedly
☐	☒	The statistical test(s) used AND whether they are one- or two-sided<br>*Only common tests should be described solely by name; describe more complex techniques in the Methods section.*
☒	☐	A description of all covariates tested
☒	☐	A description of any assumptions or corrections, such as tests of normality and adjustment for multiple comparisons
☐	☒	A full description of the statistical parameters including central tendency (e.g. means) or other basic estimates (e.g. regression coefficient) AND variation (e.g. standard deviation) or associated estimates of uncertainty (e.g. confidence intervals)
☐	☒	For null hypothesis testing, the test statistic (e.g. *F*, *t*, *r*) with confidence intervals, effect sizes, degrees of freedom and *P* value noted<br>*Give P values as exact values whenever suitable.*
☒	☐	For Bayesian analysis, information on the choice of priors and Markov chain Monte Carlo settings
☒	☐	For hierarchical and complex designs, identification of the appropriate level for tests and full reporting of outcomes
☒	☐	Estimates of effect sizes (e.g. Cohen's *d*, Pearson's *r*), indicating how they were calculated

Our web collection on statistics for biologists contains articles on many of the points above.

Software and code

Policy information about availability of computer code

Data collection	Crystal data were collected on beamlines BL19U1, BL17U1 and BL18U1 at the Shanghai Synchrotron Radiation Facility (SSRF); SPR data were collected on a Biacore T200 (Cytiva) ; RT-PCR data were collected on a CFX96 system (Bio-Rad); MD simulations data were collected using OpenMM (version 7.7).
Data analysis	HKL3000 (v716.1); Coot (v0.9.6); Phenix (v1.14-3260); PyMOL (v2.4.0); GraphPad Prism (v7.0); Origin (v2021), ccp4i (v7.1); Photoshop CC (v2017); FACSDiva software (v8.0.3), FlowJo (v10); ChenBioDraw (14.0.0.117).

For manuscripts utilizing custom algorithms or software that are central to the research but not yet described in published literature, software must be made available to editors and reviewers. We strongly encourage code deposition in a community repository (e.g. GitHub). See the Nature Portfolio guidelines for submitting code & software for further information.

Data

Policy information about availability of data

All manuscripts must include a data availability statement. This statement should provide the following information, where applicable:

- Accession codes, unique identifiers, or web links for publicly available datasets
- A description of any restrictions on data availability
- For clinical datasets or third party data, please ensure that the statement adheres to our policy

The refined structural models and corresponding structure-factor amplitudes have been deposited in the PDB database under accession codes 7Y90 [https://doi.org/10.2210/pdb7Y90/pdb] (BCL-2-cp1), 7Y8D [https://doi.org/10.2210/pdb7Y8D/pdb] (BCL-XL-cp1), 7Y99 [https://doi.org/10.2210/pdb7Y99/pdb] (BCL-XL-cp2),

7YA5 [https://doi.org/10.2210/pdb7YA5/pdb] (BCL-2-G101V-cp1), 7YAA [https://doi.org/10.2210/pdb7YAA/pdb] (BCL-XL-cp3) and 7YB7 [https://doi.org/10.2210/pdb7YB7/pdb] (BCL-2-12M-cp2). All the structures cited in this work are also available under accession codes 1MAZ [https://doi.org/10.2210/pdb1MAZ/pdb], 2XA0 [https://doi.org/10.2210/pdb2XA0/pdb], 6O0K [https://doi.org/10.2210/pdb6O0K/pdb], 1GJH [https://doi.org/10.2210/pdb1GJH/pdb], 3ZLR [https://doi.org/10.2210/pdb3ZLR/pdb], 4QVE [https://doi.org/10.2210/pdb4QVE/pdb], 2YXJ [https://doi.org/10.2210/pdb2YXJ/pdb], and 1AF3 [https://doi.org/10.2210/pdb1AF3/pdb]. Source data are provided with this paper. The MD simulation parameter files are available as a zipped folder named Supplementary Data 1.

Human research participants

Policy information about studies involving human research participants and Sex and Gender in Research.

Reporting on sex and gender	N/A
Population characteristics	N/A
Recruitment	N/A
Ethics oversight	N/A

Note that full information on the approval of the study protocol must also be provided in the manuscript.

Field-specific reporting

Please select the one below that is the best fit for your research. If you are not sure, read the appropriate sections before making your selection.

[x] Life sciences [ ] Behavioural & social sciences [ ] Ecological, evolutionary & environmental sciences

For a reference copy of the document with all sections, see nature.com/documents/nr-reporting-summary-flat.pdf

Life sciences study design

All studies must disclose on these points even when the disclosure is negative.

Sample size	Experiments were performed three times independently unless indicated. Choice of sample sizes guided by established precedents from leading works in the field. [see for example Birkinshaw et al. Nat Commun. 2019 Jun 3;10(1):2385.]
Data exclusions	No data exclusion.
Replication	All experiments were repeated at least twice with similar results. The number of biological replicates is stated in the figure legends.
Randomization	No experimental grouping requiring randomization was performed. The positions of samples on multi-well plates were different among individual experiments to minimize the systematic errors.
Blinding	N/A (data were automatic readouts of the instruments).

Reporting for specific materials, systems and methods

We require information from authors about some types of materials, experimental systems and methods used in many studies. Here, indicate whether each material, system or method listed is relevant to your study. If you are not sure if a list item applies to your research, read the appropriate section before selecting a response.

Materials & experimental systems

n/a	Involved in the study	
[ ]	[x]	Antibodies
[ ]	[x]	Eukaryotic cell lines
[x]	[ ]	Palaeontology and archaeology
[x]	[ ]	Animals and other organisms
[x]	[ ]	Clinical data
[x]	[ ]	Dual use research of concern

Methods

n/a	Involved in the study	
[x]	[ ]	ChIP-seq
[ ]	[x]	Flow cytometry
[x]	[ ]	MRI-based neuroimaging

Antibodies

Antibodies used	n our study, the primary antibodies against BCL-2 (Abcam, ab194583), BCL-XL (Abcam, ab32370), anti-Flag (Sigma, F1804) Cytochrome C (beyotime AC909), BAX (beyotime AB026), BAK (beyotime AB016) were used as 1:1000 dilution and α-Tubulin (Abcam,

ab7291), or β-Actin (Abcam, ab8227) were used as 1:3000 dilution in the western blotting. The secondary antibodies anti-Rabbit IgG (Abcam, ab6721) or Goat anti-Mouse IgG (Abcam, ab6789) were used as 1:5000 dilution in the western blotting.

Validation

Antibodies were validated for the indicated use by the manufacturer available on their websites:
1. Antibody against BCL-2
https://www.abcam.com/Bcl-2-antibody-ab194583.html
2. Antibody against BCL-XL
https://www.abcam.com/bcl-xl-antibody-e18-ab32370.html
3. anti-Flag
https://www.sigmaaldrich.cn/CN/zh/product/sigma/f1804
4. Anti-alpha Tubulin antibody
https://www.abcam.com/alpha-tubulin-antibody-dm1a-loading-control-ab7291.html
5. Anti-beta Actin antibody
https://www.abcam.com/beta-actin-antibody-ab8227.html
6. Goat Anti-Rabbit IgG H&L (HRP)
https://www.abcam.com/goat-rabbit-igg-hl-hrp-ab6721.html
7. Goat Anti-Mouse IgG H&L (HRP)
https://securedrtest.abcam.com/goat-mouse-igg-hl-hrp-ab6789.html
8. Antibody against Cytochrome C
https://www.beyotime.com/product/AC909.htm
9. Antibody against BAX
https://www.beyotime.com/product/AB026.htm
10. Antibody against BAK
https://www.beyotime.com/product/AB016.htm

Eukaryotic cell lines

Policy information about cell lines and Sex and Gender in Research

Cell line source(s)

Jurkat (https://www.atcc.org/products/tib-152), El4(https://www.atcc.org/products/tib-39), Daudi (https://www.atcc.org/products/ccl-213), RS4;11 (https://www.atcc.org/products/crl-1873), A549 (https://www.atcc.org/products/ccl-185), hepG2 (https://www.atcc.org/products/hb-8065), MGC-803 (https://www.beyotime.com/product/C6582.htm), PANC-1 (https://www.atcc.org/products/crl-1469).

Authentication

Cell authentication was performed using the short tandem repeats (STR) method by the respective vendors.

Mycoplasma contamination

All cell lines tested negative for Mycoplasma contamination.

Commonly misidentified lines
(See ICLAC register)

No commonly misidentified cell lines were used in this study.

Flow Cytometry

Plots

Confirm that:

☒ The axis labels state the marker and fluorochrome used (e.g. CD4-FITC).

☒ The axis scales are clearly visible. Include numbers along axes only for bottom left plot of group (a 'group' is an analysis of identical markers).

☒ All plots are contour plots with outliers or pseudocolor plots.

☒ A numerical value for number of cells or percentage (with statistics) is provided.

Methodology

Sample preparation

Jurkat cells were seeded to 6-well plates at a density of 4x105 cells per well. Cells were incubated in the presence of DMSO, venetoclax at 2.5 µM, cp1-TAT at 30 µM, cp2-TAT at 30 µM, or cp3-TAT at 20 µM in serum-free RPMI medium for 4 h. After being incubated totally for 24 h, cells were washed once with PBS and stained with 100 µL binding buffer containing 5 µL Annexin V-FITC and 10 µL PI for 15 min, followed by addition of 400 µL binding buffer.

Instrument

BD FACSAria Fusion was used for data collection.

Software

The FACSDiva software (v8.0.3) was used in data collection and the FlowJo (v10) was used in data analysis.

Cell population abundance

We only conducted the apoptosis experiment of a single cell line with flow cytometry.

Gating strategy

DMSO and TAT treated cells served as blank controls, while venetoclax treated cells served as the positive control, which together defined the Q1 and Q3 regions that respectively represent cells with red and green fluorescence.

☒ Tick this box to confirm that a figure exemplifying the gating strategy is provided in the Supplementary Information.
